# Comparison of patient reported dry eye symptoms as evaluated by the ocular surface disease index and symptom assessment

**DOI:** 10.4102/phcfm.v17i1.4861

**Published:** 2025-05-21

**Authors:** Solani D. Mathebula, Percy R. Khosa, Mmathabo M. Maleswene

**Affiliations:** 1 Department of Optometry, Faculty of Health Sciences, University of Limpopo, Polokwane, South Africa

**Keywords:** dry eye disease, questionnaire, ocular surface disease index, symptom assessment in dry eye, dry eye symptoms, ocular surface disease

## Abstract

**Background:**

Dry eye disease (DED) is a growing public health problem because of excessive time spent on digital devices as a risk factor. The diagnosis of DED should not only be based on the objective clinical measures but also on symptoms reported by patients. Clinicians should not rely on establishing symptoms using open verbal questioning during the case history but should quantify patient symptoms using a validated questionnaire.

**Aim:**

To compare the patient-reported symptoms of DED as assessed using the Ocular Surface Disease Index (OSDI) and Symptom Assessment in Dry Eye (SANDE).

**Setting:**

An optometry clinic.

**Methods:**

Ocular Surface Disease Index and SANDE questionnaires were administered to 40 participants. Participants completed all questionnaires in a non-randomised order. The correlation between the questionnaires’ scores was determined and the Bland–Altman plot was used to assess the agreement between the two questionnaires.

**Results:**

The mean scores for OSDI and SANDE were 37.85 ± 23.79 and 38.83 ± 26.39, respectively. The Spearman correlation between the two questionnaires was 0.7, *p* < 0.01. The mean difference between OSDI and SANDE was −0.97 (95% Confidence Interval [CI]: [−7.04 to 5.09]). The Bland–Altman analysis between OSDI and SANDE showed a mean clinical difference (bias) of –0.98.

**Conclusion:**

The SANDE questionnaire can be used as a dry eye symptom assessment as it is highly correlated to the OSDI questionnaire.

**Contribution:**

Because the OSDI and SANDE questionnaires showed a significant correlation and negligible score differences, this suggests that the SANDE questionnaire has the potential to provide clinicians with a short and quick measure of DED symptoms.

## Introduction

Dry eye disease (DED) is a common multifactorial disease of the ocular surface characterised by the loss of homeostasis of the tear film and is accompanied by ocular symptoms.^1,2,3,4,5,6^ Physiologically, homeostasis refers to self-regulatory processes by which biological systems maintain internal stability while adjusting to changing external environments.^1^ The ocular symptoms of DED include ocular pain, burning sensation, dryness, grittiness, itching, irritation, photophobia and foreign body sensation, and are accompanied by fluctuating vision with blurred or double vision.^2,3,4,5,6^ These symptoms affect quality of life by disturbing crucial daily activities such as work, reading, driving, using visual display units and watching television.^6,7,8,9^ Dry eye disease is considered a major public health issue, affecting millions of people globally, and is one of the most frequent reasons for ophthalmic consultations.^1,3^

As there is no ‘gold standard’ diagnostic test for DED, the diagnosis should be made on the combination of dry eye symptoms and clinical signs.^10,11^ Patients are considered to have DED if there are subjective symptoms and at least one clinical sign, such as decreased tear break-up time or notable epithelial damage, which are positive markers of homeostasis.^11^ Generally, there is a weak or poor correlation between subjective dry eye symptoms and objective clinical signs because of a lack of positive clinical signs in patients with dry eye who have mild to moderate symptoms.^10,11,12,13,14^

Because of poor repeatability of findings on clinical evaluation of DED, clinicians should also base their assessment of DED on the results of validated questionnaires.^6,7,10^ The questionnaires are the most repeatable diagnostic instrument for symptom assessment, as they are generally more predictive of DED diagnosis as compared to the clinical signs and play a crucial role in the diagnosis and management of DED.^10^

The most widely available standardised questionnaires for the evaluation of DED symptoms include the Ocular Surface Disease Index (OSDI), Dry Eye Questionnaire (DEQ-5), 25-Item National Eye Institute Visual Function Questionnaire (NEI VFQ-25), Dry Eye Questionnaire for Dry Eye Epidemiology Projects (DEEP), Impact of Dry Eye on Everyday Life (IDEEL), Standardised Patient Evaluation of Eye Dryness (SPEED), and Symptom Assessment in Dry Eye (SANDE).^11,15,16,17,18,19,20^ However, these questionnaires vary in length and commonly involve a series of different rating scale type items that are combined to produce a total raw score. Therefore, clinicians should consider the characteristics of each relevant questionnaire during dry eye assessment.

The OSDI questionnaire is the most frequently used questionnaire, which is a 12-item instrument to assess subjective dry eye symptoms. It has three subscales: ocular symptoms with three questions, vision-related functions with six questions and environmental triggers with three questions.^20,21,22,23,24,25^ The OSDI requires approximately 5 min for the patient to complete, and the score ranges between 0 and 100, where the higher the score, the greater the severity of symptoms. It is a valid and reliable instrument for measuring the severity of DED as it can discriminate between normal, mild to moderate and severe DED, and between normal individuals and patients with DED, as defined by both the physician’s assessment and a composite disease severity score.^20,21,22,23,24,25^

The SANDE is comprised of two questions to quantify dry eye symptom frequency and severity on a 100 millimetre horizontal linear visual analogue scale (VAS).^26,27^ The measurement of SANDE requires respondents to answer two questions: responses on the symptom frequency range from left to right, ‘rarely’ to ‘all the time’, and the symptom severity from ‘very mild’ to ‘very severe’ on a VAS scale. It has been shown that both the OSDI and SANDE tests are reliable and valid measures of dry eye symptom.^21,27^ The final SANDE score of each response from the far left to right in millimetres is scored by multiplying the frequency score by the severity score and obtaining the square root.^26^ However, the SANDE questionnaire has a weak correlation with clinical tests (such as corneal staining) but a strong correlation for monitoring the patients’ therapeutic response.^26,27^

Although the validity of the SANDE questionnaire has been reported,^26,27^ the performance of this questionnaire compared to the mostly used dry eye symptom questionnaire has not been well studied in clinical populations. The purpose of this study was to compare the performance of the SANDE with the OSDI in participants with DED while exploring the potential use of the SANDE questionnaire, as it will allow clinicians to quickly assess dry eye symptoms.

## Research methods and design

### Study design

This was a clinical-based evaluation of diagnostic tests.

### Setting

Measurements were collected at the optometric clinic.

### Study population and sampling strategy

Tests were performed on participants aged 18 years and above who had a previous diagnosis of DED and were representative of the subset of DED seen in the optometry clinic.

**FIGURE 1:**
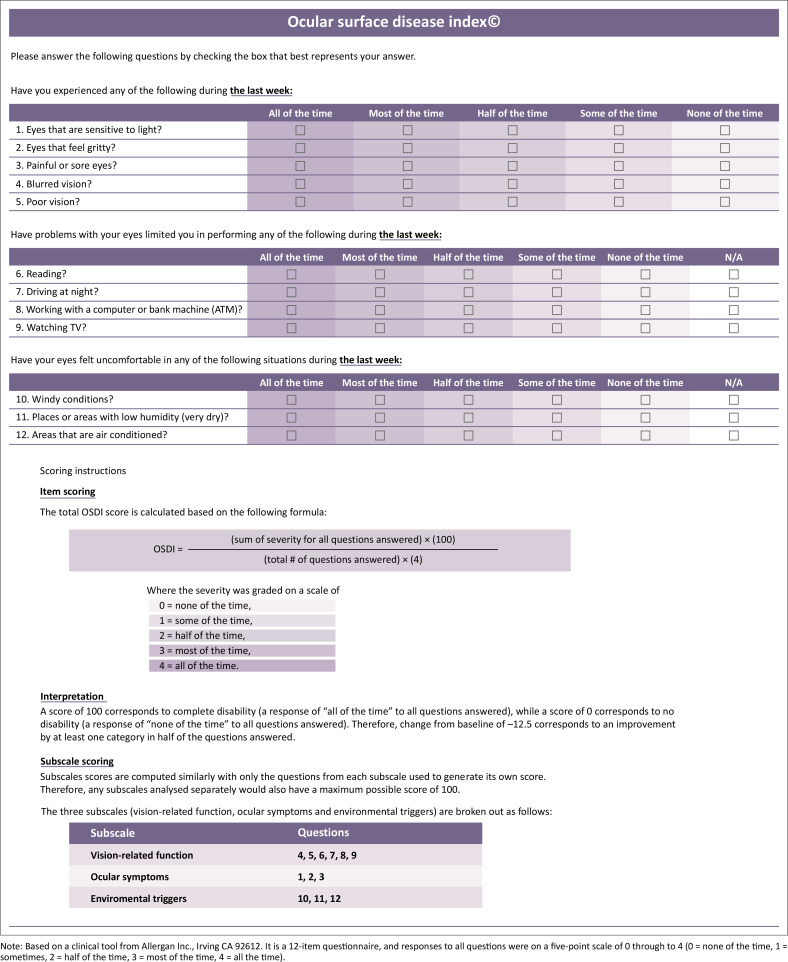
Ocular surface index questionnaire.

**FIGURE 2:**
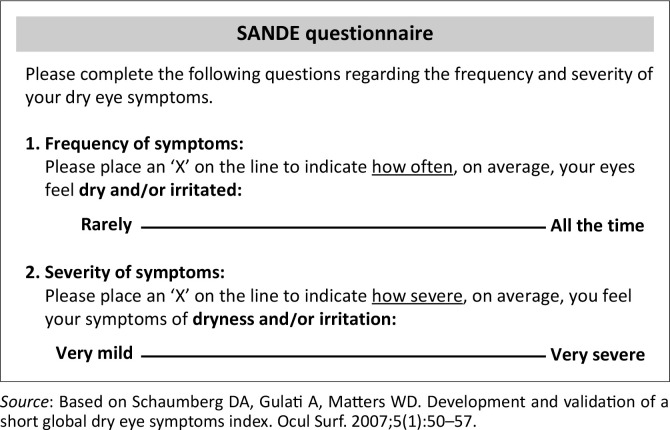
Symptom assessment in dry eye questionnaire.

This study was conducted in compliance with the tenets of the Declaration of Helsinki. Participants were purposefully recruited from patients presenting for eye examination with a diagnosis of DED at the Optometry clinic of the University of Limpopo, South Africa. A non-random sample of 40 non-contact lens-wearing young adult participants with ocular dryness symptoms were recruited to volunteer in this study. Participants were recruited based on the variety of dry eye symptoms as presented by the chief complaints. All participants were informed in detail about the benefits and risks of the study, and consent was obtained. All participants were evaluated in a single visit, and both questionnaires (see Figure 1 and Figure 2^26^) were conducted via interviews by the researchers. All researchers are trained optometrists, and there was no impact of the inter-practitioner variability in the study procedures.

Dry eye disease patients with a history of ocular disease other than the clinical sign of dry eye were excluded. Participants were included if they reported experiencing ocular dryness ‘sometimes, most of the times or all the time’ during the routine case history. Participants who had previously undergone corneal refractive surgery, had a history of eye medication known to affect the ocular surface or had a history of ocular trauma were also excluded. Corneal fluorescein staining was used as the objective selection criteria and demonstrated a corneal fluorescein stain of more than three spots and tear breakup time (TBUT) < 10 seconds.^28^

### Data collection

All participants were purposefully administered the OSDI and SANDE questionnaires. The OSDI was administered first, and the researcher remained with the participant throughout for questions and doubts but did not intervene to avoid influencing the answering process. The OSDI was populated to assess dry eye symptoms within the previous week (see Figure 1).

Participants were then classified according to the recommended OSDI cut-off values into non-dry eye symptom (OSDI < 13) and dry eye symptom (OSDI > 13).^21^ The overall OSDI score is obtained by calculating the scores using the following formula in Equation 1:


$$\text{OSDI score}=\frac{\text{Sum of OSDI scores x }25}{\text{Total number of questions answered}}$$ [Eqn 1]

The OSDI scores were then grouped to classify the participants’ dry eye symptoms as normal or no symptom (˂ 13), mild (13–22), moderate (23–32), and severe (˃ 32). Participants with an OSDI score greater than 12 points were considered positively symptomatic for DED.^24^

The SANDE questionnaire consists of two questions that quantify dry eye symptom frequency and severity within the past 2 months on a 100 mm horizontal visual analogue scale. Participants were asked to put a mark on two given lines to show the extent of their symptoms, separately in terms of frequency and severity. To simplify the procedure for participants, the extreme left of the 100 mm line indicated ‘rarely’ and the extreme right indicated ‘all the time’ for frequency or ‘very mild’ to ‘very severe’ for severity (see Figure 2^26^). Participants were asked to place a mark on the two given lines based on the extent of their symptoms. The location of the mark made by the participant was measured from left to right and recorded in millimetres. The SANDE score was obtained by multiplying the frequency score by the severity score, and taking the square root of the score to transform back to the original scale.^26,27^ However, the SANDE questionnaire does not have a designated cut-off value like the OSDI.

### Data analysis

Statistical analysis of data was performed using a Statistical Package for Social Sciences (SPSS version 27 for Windows, SPSS Inc., Chicago, IL, USA). Shapiro–Wilk test was used to assess the normality of the data.^29^ Means and standard deviations were used to describe data. The intraclass correlation coefficient (ICC) Cronbach’s alpha, which is the measure of internal consistency, was used to evaluate the reliability of the OSDI and SANDE questionnaires.^29,30^ A Bland–Altman plot was performed to calculate the differences of scores and plot them against the average scores. For all analyses, *p*-values > 0.05 were considered statistically significant. We used the paired *t*-test to calculate the mean difference between OSDI and SANDE questionnaires. A mathematical model to estimate SANDE or OSDI using OSDI or SANDE was derived via regression analysis.

### Ethical considerations

The study was conducted in compliance with the Helsinki Declaration, and informed consent was obtained from all participants. Ethical clearance to conduct the study was obtained from the University of Limpopo, Turfloop Research Ethics Committee (No. TREC/484/2023: UG). All participants were informed about the nature of the study and its consequences.

## Results

The inclusion criteria were met by 40 (25 females and 15 males) participants, aged between 20 and 28 years, with a mean and standard deviation (s.d.) of 22.4 ± 2.3 years. Both scores from the OSDI and SANDE questionnaires were normally distributed, as assessed by the Shapiro-Wilk test, *p* > 0.05. According to the OSDI score grading, 8 (19.5%), 10 (25%) and 18 (45%) participants had severe, moderate and mild dry eye symptoms, respectively. There were 5 (12.5%) participants who did not have dry eye symptoms according to the OSDI. The descriptive statistics of the comparison of OSDI and SANDE questionnaires are presented in Table 1. The scores ranged from 0 to 85 and 0 to 82 for OSDI and SANDE, respectively, and showed no statistically significant difference, *p* = 0.43.

**TABLE 1:** Descriptive statistics of the means for the dry eye symptoms assessed using ocular surface disease index and symptom assessment in dry eye.

Questionnaire	Mean ± SD	Median	Min	Max	Skewness	Kurtosis	*p*
OSDI	37.85 ± 23.8	29.5	0.0	85	0.4	−1.0	0.43
SANDE	38.83 ± 26.4	43.0	0.0	82	0.2	−1.5	-

Note: The findings are nonrandomised scores.

OSDI, ocular surface disease index; SANDE, symptom assessment in dry eye; s.d., standard deviation.

**FIGURE 3:**
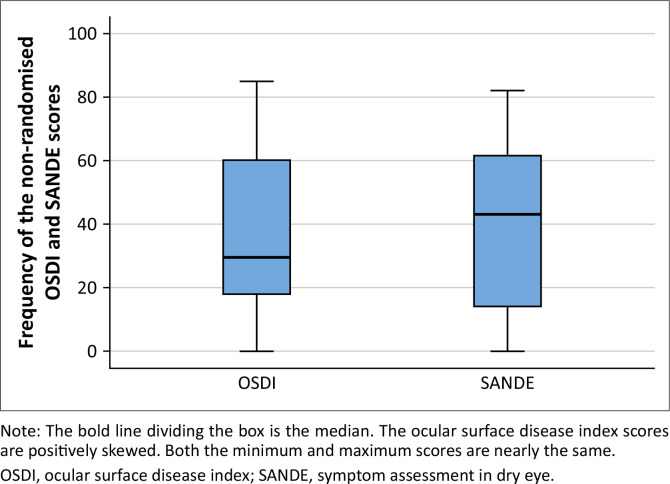
Box plots for the nonrandomised ocular surface disease index and symptom assessment in dry eye questionnaire’ scores.

Figure 3 shows the boxplots for the OSDI and SANDE scores. There were no outliers in the data. The median is shown by the horizontal line inside the box. The lines extending from the top and the bottom of the box are whiskers representing the minimum and the maximum values. The scores for the OSDI were positively skewed (0.4). This is shown by the median being closer to the bottom of the box.

**FIGURE 4:**
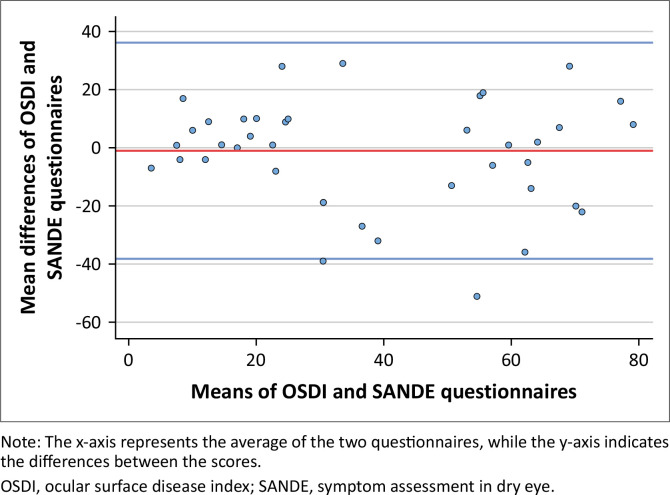
The Bland–Altman plot comparing ocular surface disease index and symptom assessment in dry eye scores.

The Spearman correlation coefficient (*r*) between the OSDI and SANDE scores was 0.7, *p* < 0.001. There is a strong positive correlation between OSDI and SANDE raw scores. The mean difference was −0.98 ± 18.98 (95% CI: −7.04 to 5.09). The Bland–Altman plot, which is a scatter diagram, shows the clinical agreement between OSDI and SANDE questionnaires where the mean difference between scores of OSDI and SANDE are plotted on the *y*-axis while the means of the two questionnaires are then plotted on the *x*-axis (see Figure 4). The red middle line represents the mean difference (bias) of –0.98 units. The two outer lines are the upper and lower limits of agreements (LoA), which represent the ±1.96 SD of the mean difference. They depict the intervals that include 95% of all the differences.

## Discussion

In this study, we found that the mean difference was small between the means of the OSDI and SANDE questionnaires, and there was no statistically significant difference in the questionnaires, *p* > 0.05. The SANDE scores were found to correlate well with those from the OSDI questionnaire (*r* = 0.7, *p* < 0.01). The strong correlation shown between OSDI and SANDE scores, together with their clinically negligible mean differences, suggests that the SANDE questionnaire scores are comparable to those from the OSDI. Cronbach’s alpha is a measure of internal consistency and was used in this study to evaluate the reliability of the OSDI and SANDE questionnaires.^29^

Amparo et al.^31^ compared OSDI and SANDE questionnaires in patients with dry eye and found significantly correlated results (*r* = 0.64, *p* < 0.01) at the baseline visit and moderate correlation (*r* = 0.47, *p* < 0.0001) at follow-up. They concluded that the clinically negligible differences suggest that the SANDE scores are equivalent to those from the OSDI questionnaire.

Martin and EMO Research Group^32^ conducted a study to compare the OSDI and SANDE in a sample of patients with and without dry eye symptoms. The authors found that the scores from the SANDE questionnaire were different from those of the OSDI questionnaire. They concluded that the results of both questionnaires cannot be used interchangeably in clinical practice. Kheirkhah et al.^33^ performed a randomised study to assess clinical changes after intraductal meibomian gland probing on 42 patients using OSDI and SANDE questionnaires. They found that the SANDE mean score was higher than the OSDI one. Chen et al.^34^ examined the relationship between tear osmolarity and dry eye symptoms in women using oral contraceptive pills. The dry eye symptoms were assessed using the OSDI and SANDE questionnaires. Overall, OSDI correlated well with both SANDE severity (*r* = 0.53, *p* < 0.0001) and frequency (*r* = 0.63, *p* > 0.0001). However, Trave-Huarte and Wolffsohn^35^ found significantly lower SANDE scores than OSDI scores in dry eye patients.

The Bland–Altman plot showed a mean difference of –0.9 (95% CI: −7.0 to 5.0) between OSDI and SANDE scores. The analysis showed that SANDE score is 12 points higher than OSDI. This result is in agreement with the conclusion reported by Chen et al.^34^ that the SANDE questionnaire scored symptoms 14 points higher than the OSDI. Although 12 points would be, in fact, clinically significant, Miller et al.^24^ reported that such a difference is clinically negligible if the difference would remain the same in the follow-up. Since 95% of the data points lie within the ±1.96 SD of the mean difference, this indicates that the two methods are producing comparable results.

The cause for such differences could be that OSDI consists of 12 questions, and it yields scores from 0 to 100.^36,37,38^ The OSDI questionnaire requires patients to read, understand and then respond to 12 questions, while SANDE is a short questionnaire. Although OSDI is a validated questionnaire, it measures only the frequency of dry eye symptoms, while SANDE measures both the frequency and severity of symptoms. In addition, the OSDI does not address all dry eye symptoms, such as foreign body sensation.

The study has some limitations. The recruited participants were sampled from a clinical population. This may affect the applicability of these results to other participants wearing contact lenses and those who had ocular surgical procedures. The sample size may not be ideal to give conclusive results, and these two questionnaires might not be the most accurate. The study did not have a control group.

Although several questionnaires to assess dry eye symptoms are available, a short and easily comprehensible questionnaire that can monitor symptom frequency and severity is needed. Based on the results of this study, we think these two questionnaires can complement each other to outperform their accuracy and precision when used independently.

## Conclusion

The performance of the SANDE questionnaire is comparable to the OSDI in discriminating symptoms of dry eye since the overall scores are highly correlated with negligible mean differences. This suggests that the SANDE questionnaire can be used clinically and in research studies as a quick assessment of DED symptoms. Future studies should be conducted to examine the performance of these two DED questionnaires in clinical trials.
